# Effect of Different Functional Food Supplements on the Gut Microbiota of Prediabetic Indonesian Individuals during Weight Loss

**DOI:** 10.3390/nu14040781

**Published:** 2022-02-13

**Authors:** Ingrid S. Surono, Abraham Simatupang, Pratiwi D. Kusumo, Priyo Waspodo, Sanne Verbruggen, Jessica Verhoeven, Koen Venema

**Affiliations:** 1 Food Technology Department, Faculty of Engineering, Bina Nusantara University, Jakarta 11480, Indonesia; isurono@binus.edu (I.S.S.); priyowaspodo@yahoo.com (P.W.); 2 Faculty of Medicine, Universitas Kristen Indonesia, Jakarta 13630, Indonesia; abraham.simatupang@uki.ac.id (A.S.); pratiwi.kusuma@uki.ac.id (P.D.K.); 3 Centre for Healthy Eating & Food Innovation, Maastricht University—Campus Venlo, 5928 SZ Venlo, The Netherlands; s.verbruggen@maastrichtuniversity.nl (S.V.); jessica.verhoeven@maastrichtuniversity.nl (J.V.)

**Keywords:** taro, probiotic, *L. plantarum* IS-10506, beetroot, gut microbiota, prediabetes

## Abstract

The gut microbiota has been shown in recent years to be involved in the development and severity of type 2 diabetes (T2D). The aim of the present study was to test the effect of a 2-week functional food intervention on the gut microbiota composition in prediabetic individuals. A randomized double-blind, cross-over trial was conducted on prediabetic subjects. Fifteen volunteers were provided products made of: (i) 50% taro flour + 50% wheat flour; (ii) these products and the probiotic *L. plantarum* IS-10506; or (iii) these products with beetroot adsorbed for a period of 2 weeks with 2 weeks wash-out in between. Stool and blood samples were taken at each baseline and after each of the interventions. The gut microbiota composition was evaluated by sequencing the V3–V4 region of the 16S rRNA gene and anthropometric measures were recorded. The total weight loss over the entire period ranged from 0.5 to 11 kg. The next-generation sequencing showed a highly personalized microbiota composition. In the principal coordinate analyses, the samples of each individual clustered closer together than the samples of each treatment. For six individuals, the samples clustered closely together, indicating a stable microbiota. For nine individuals, the microbiota was less resilient and, depending on the intervention, the beta-diversity transiently differed greatly only to return to the composition close to the baseline during the wash-out. The statistical analyses showed that 202 of the total 304 taxa were significantly different between the participants. Only *Butyricimonas* could be correlated with taro ingestion. The results of the study show that the highly variable interindividual variation observed in the gut microbiota of the participants clouded any gut microbiota modulation that might be present due to the functional food interventions.

## 1. Introduction

The increase of type 2 diabetes (T2D) worldwide is occurring at a dramatic speed. By the year 2030, of a projected world population of 8.5 billion [1], 360 million people are predicted to have T2D [2]. An increase in energy intake and a decrease in energy expenditure are the leading commonly accepted causes of obesity associated with T2D and also metabolic syndrome and cardiovascular disease. However, the gut microbiota has recently also been shown to play an important role [3,4,5,6]. Both the composition and/or the activity of the gut microbiota can be changed using functional food ingredients. The vital role that food plays in T2D, both for prevention and treatment, needs proper attention. For instance, of particular importance is the development of dietary components that positively influence postprandial glycaemia and because of the lowered blood glucose, may have the potential to reduce the impact of T2D. In addition, the effects of food ingredients on T2D through the modulation of the composition and/or activity of the gut microbiota need to be considered.

Indonesia is particularly rich in plant biodiversity. This includes a variety of local tubers. Despite widespread use in the past and their anecdotal and potential functional properties, these tubers are currently underutilized. One of these interesting tubers is Cocoyam or taro, which belongs to the monocotyledonous family Araceae (the aroids) [7,8,9]. The taro tuber was an important ethnic root crop throughout Asia and was also related to the culture of regions. Hence, taro was considered to be very important for community life in the past [10]. As it adapts well to different agro-climatic conditions, it was used as a staple crop in various parts of the humid tropics and sub-tropics [7,8,9]. Kreike et al. [11] reported that Indonesia has the highest taro diversity in the world. Taro tubers are cultivated in areas in Borneo, Java, Sumatra, and Sulawesi [12] although nowadays the cultivation is widely naturalized, being also available in Africa and the Americas. Taro was traditionally used as an alternative carbohydrate source to reduce the dependence on rice. Recently, we have shown in several rat models of diabetes that taro, or its purified starch, has an effect on the gut microbiota composition [13,14]. Moreover, using a sophisticated, dynamic in vitro model of the upper gastrointestinal tract, we have shown that a large portion of taro starch is resistant to digestion and can reach the colon—and its associated gut microbiota—as resistant starch [15,16].

Probiotics are defined as “life microorganisms, which when administered in adequate amounts, have a beneficial effect on the host” [17]. Probiotic strains, amongst which are members from the family Lactobacillaceae and the genus *Enterococcus*, have been isolated from dadih, a traditional fermented buffalo milk produced in West Sumatra [18]. Previous research has shown that dadih consumption reduces adiposity, weight gain, and adiposity inflammation in high fat-induced obese rats [19]. The microbes present in dadih, amongst which is the probiotic *Lactiplantibacillus plantarum* (L.; formerly *Lactobacillus plantarum*) strain IS-10506 [18,20,21,22], may contribute to this beneficial effect.

Beet juice has been shown to have a high total antioxidant capacity and total polyphenol content. This is believed to be caused because beet juice is rich in bioactive compounds such as phenolic acids, flavonoids, and betalains [23,24]. Polyphenols are a class of compounds including flavonoids, phenolic acids, proanthocyanidins, and tannins amongst others. These bioactives have been suggested to be able to modify postprandial (hyper)glycaemia [25,26] by several mechanisms. These include inhibiting carbohydrate digestion, reducing glucose absorption in the intestines, the stimulation of insulin release from pancreatic β-cells, the modulation of hepatic glucose output, the activation of insulin receptors, and/or the modulation of glucose uptake in insulin-sensitive cells [27,28]. As polyphenols are not well-absorbed in the small intestine, partly because they can be glycosylated, they can reach the colon where they have the potential to modulate the composition and/or activity of the gut microbiota. Therefore, beet juice, with its reported content of polyphenols, is an interesting food model to investigate any influence on the glycemic response either by direct mechanisms such as the inhibition of glucose uptake or by indirect action affecting insulin sensitivity, whether or not through the modulation of the composition and/or activity of the gut microbiota.

The aim of the current study was to study the effect of taro starch alone or in combination with the probiotic *L. plantarum* IS-10506 or beetroot on the gut microbiota of prediabetic Indonesian individuals during weight loss.

## 2. Materials and Methods

### 2.1. Study Design and Population

This study was a community-based, double-blind, randomized controlled cross-over clinical trial involving 15 subjects with a diagnosis of prediabetes or early stage type 2 diabetes with an age range of 33–62 years comprising 6 men and 9 women (Table 1). In the early stages of the research, 136 volunteers were recruited and selected based on the inclusion criteria as follows: fasting blood glucose of 100–125 mg/dL; random blood glucose of 140–199 mg/dL; BMI 25–27 kg/m^2^; and subjects who did not consume antibiotics and probiotics two weeks before the start of the intervention. The subjects were willing to undergo a weight loss program and communicate intensively with the intervention staff. They consumed fruit, vegetables, snacks, and drinks 3 times a day, which was provided for 14 days in each of the 4 treatment arms. People with hyperlipidemia, who consumed metformin, expecting and lactating mothers and women with hormonal constipation, as well as individuals with an established history of cardiovascular or other metabolic, hormonal, liver, and kidney diseases were excluded.

Sample size calculations on the basis of changes in the gut microbiota composition are difficult because there are conflicting data reported with respect to the different measures. The sample size calculation in this study was based on the Bacteroidetes to Firmicutes ratio, which in several prior studies (e.g., [29]) has been shown to correlate with obesity but not in others. G*Power 3.9.1.7 [30] was used to calculate the sample size based on an effect size dz of 0.1, an α error probability of 0.05, a power of 0.95, and a mean difference of 0.1 with an SD of 0.01. That led to a sample size of 10. We decided to include 18 volunteers, expecting a few drop-outs.

Out of 136 subjects, 18 subjects were selected, of which 15 completed the study. The protocol was approved by the Research Ethics Committee of the University of Indonesia (dossier No.KET-329/UN2.F1/ETIK/PPM.00.02/2019) and written informed consent was obtained from the subjects. The work described was carried out in accordance with The Code of Ethics of the World Medical Association (Declaration of Helsinki) for experiments involving humans. Informed consent was obtained from the individuals prior to the start of the run-in period.

Stool and blood samples were collected before and after two-week interventions for each type of treatment (control (run-in), taro, taro and probiotic, taro and beet juice in that order). Treatments were provided for a two-week period with a two-week wash-out period in between each treatment. The total study duration was 14 weeks. Apart from fruit and vegetables, the (starch-containing) foods provided were in the form of noodles, bread, flakes, biscuits, cookies, and chocolate drinks with the main ingredient being 50% taro flour and 50% wheat flour for the three treatment periods and 100% wheat flour for the control period. During the taro + probiotic period, the volunteers also received 10^8^ CFU of the probiotic *L. plantarum* IS-10506 twice daily in the form of microencapsulated cells [18]. During the taro + beetroot period, 6 g of beetroot powder was mixed with the chocolate drinks. During the wash-out period, the subjects reverted to their habitual diet but with the recommendations by the dieticians in the weight loss program on calorie restriction. The subjects recorded the foods they consumed in a food diary.

The study describing the changes in the blood parameters is in preparation. In this paper, we have focused on the microbiota composition. Microbiota analyses were carried out to determine the gut microbiota profiles associated with the 14-day intervention after each treatment by taking stool samples prior to the start of each intervention (4 baseline samples) and at the end of the 4 intervention periods, providing 8 samples in total.

### 2.2. DNA Isolation and Sequencing of the V3–V4 Region of the 16S rRNA Gene

DNA isolation and sequencing of the barcoded amplicons of the V3–V4 region of the 16S rRNA gene were performed according to the established protocols provided by Illumina (Illumina, Eindhoven, The Netherlands) as previously described by us [13,14]. The sequencing was performed using the Illumina MiSeq system (San Diego, CA, USA). QIIME 2 software was used for the analyses of the raw sequences. The sequences were classified using Greengenes (version 13.8) as a reference 16S rRNA gene database [13,14].

### 2.3. Statistical Analyses

Correlations between the amplified sequence variants (ASVs) and the different categorical variables such as sex, sampling site, or intervention were investigated using the non-parametric Kruskal–Wallis test corrected with the Benjamini–Hochberg false discovery rate (FDR) for multiple comparisons by using the software package R (3.5.3) (R Core Team, http://www.R-project.org/; accessed on 11 November 2021) in RStudio. Non-parametric Spearman’s rank-order correlations were obtained between the ASVs and continuous variables such as age and weight. The *q*-values (adjusted *p*-values after the FDR) were considered significantly different at a strict cut-off of *q* < 0.05.

## 3. Results

After screening, 18 participants were included, of which 15 individuals completed the 4 interventions and were included in the gut microbiota analysis (Table 1). Based on their plasma IL-6 concentrations, they showed a mild inflammation (manuscript in preparation) as can be expected in overweight people. The HOMA-IR at the start of the study was on average 6 with all individuals > 3.84 except one (manuscript in preparation). Although different cut-offs have been defined for metabolic syndrome, in all cases a value of ≥ 3.8 is considered to be indicative of metabolic syndrome [31]. Alongside their weight loss program, the participants followed interventions with a 50:50 mix of taro/wheat flour alone, or taro/wheat flour with the probiotic *L. plantarum* IS-10506, or taro/wheat flour with beetroot adsorbed compared with wheat flour alone. The samples for the gut microbiota analysis were taken at the start of each intervention (including the run-in period) and after two weeks of each intervention and analyzed for the composition using the sequencing of the amplicons of the 16S rRNA V3–V4 region.

**Figure 1:**
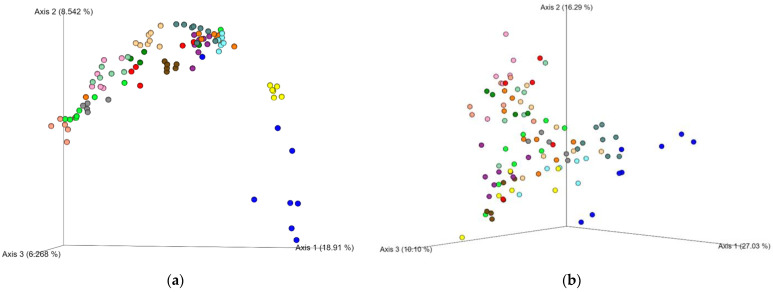
Unweighted (**a**) and weighted UniFrac (**b**) for all samples color-coded by the 15 different individuals.

**Figure 2:**
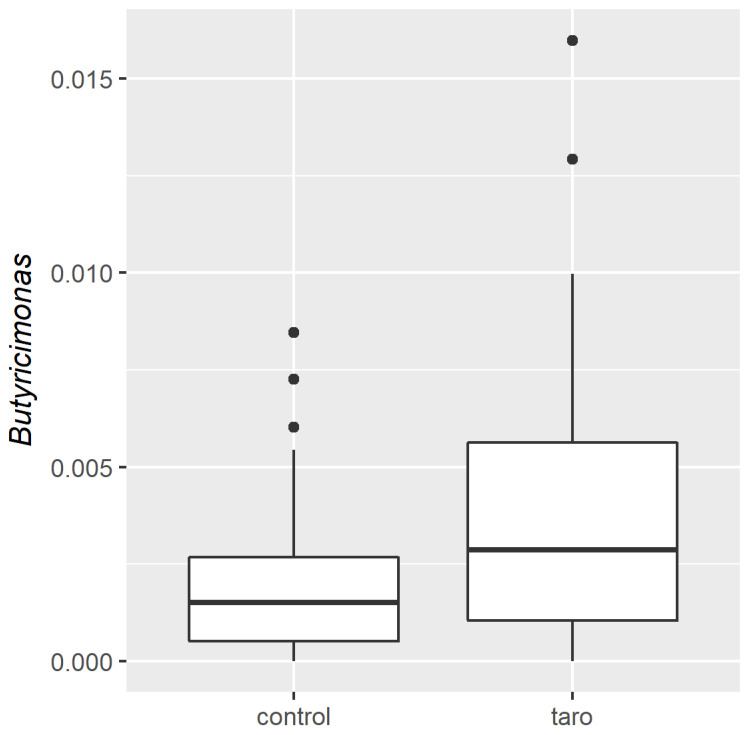
Difference in *Butyricimonas* between the control (including four baseline samples and the end sample after control intervention) and taro feeding (end samples after taro, taro + probiotic, and taro + beetroot).

Figure 1 shows the unweighted (Figure 1a) and weighted (Figure 1b) UniFrac β-diversity. Figure 1a shows a strong individual microbiota composition for the different participants. This large interindividual variation clouded any differences provided by the intervention and only the genus *Butyricimonas* was significantly different (*q* = 0.046) between the conditions with and without taro (alone, combined with the probiotic, or beetroot; Figure 2; see Supplementary Figure S1 for the individual interventions). The relative abundance of *Butyricimonas* was on average 0.26% in the full dataset with an average of 0.19% in the baseline samples and an average of 0.39% in the taro samples. Supplementary Figure S2 shows the relative abundance of the major taxa in the population.

**Figure 3:**
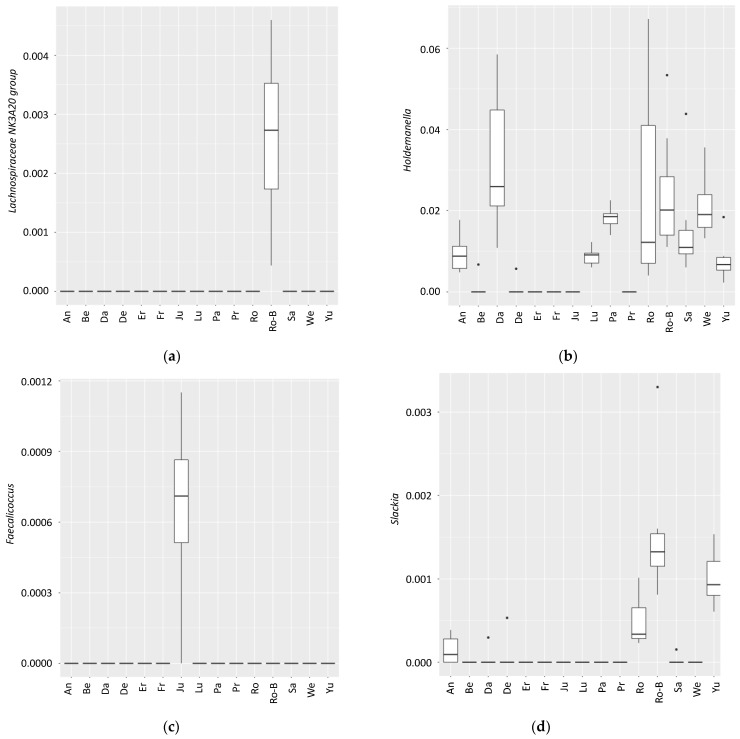

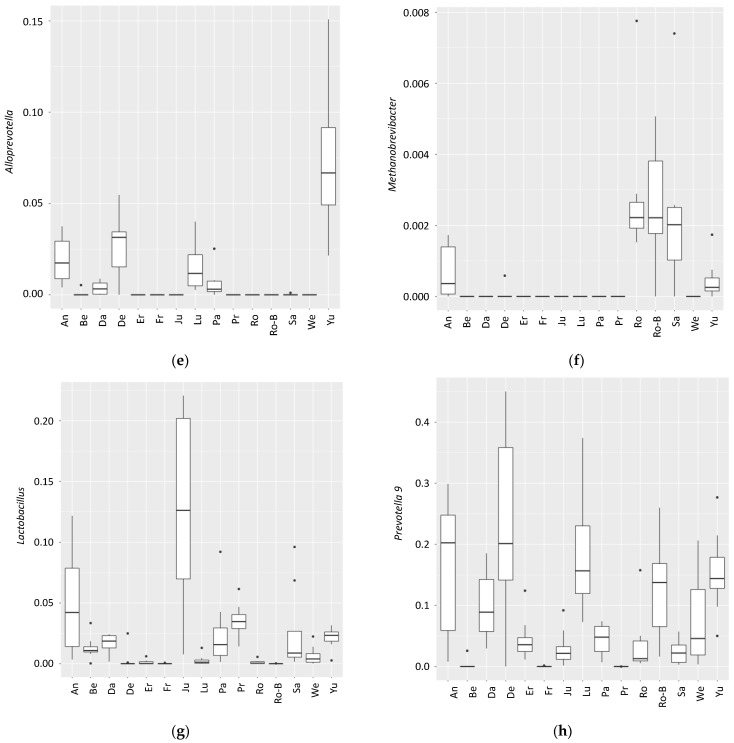
Differences in several taxa between individuals indicating the high interindividual variation in gut microbiota composition. *Lachnospiraceae NK3A20 group* ((**a**); *q*-value = 6.9 × 10^−17^); *Holdemanella* ((**b**); *q*-value = 4.6 × 10^−14^); *Faecalicoccus* ((**c**); *q*-value = 5.5 × 10^−14^); *Slackia* ((**d**); *q*-value = 7.4 × 10^−14^); *Alloprevotella* ((**e**); *q*-value = 8.3 × 10^−14^); *Methanobrevibacter* ((**f**); *q*-value = 7.6 × 10^−12^); *Lactobacillus* ((**g**); *q*-value = 2.2 × 10^−11^); *Prevotella 9* ((**h**); *q*-value = 1.8 × 10^−10^).

The strong interindividual variation was reflected in the fact that 202 of the total 304 operational taxonomic units (OTUs) were significantly different (*q* < 0.05) between the individuals when tested with the Kruskal–Wallis analysis and Benjamini–Hochberg FDR correction. Figure 3 shows the top hits (with the highly significant *q*-values in the legend).

The samples were from two different sampling sites (two universities). A total of 89 taxa were significantly different between the two sampling sites (not shown), mostly due to the high interindividual differences (Figure 3). No correlations were found for sex, age, or weight loss.

**Figure 4:**
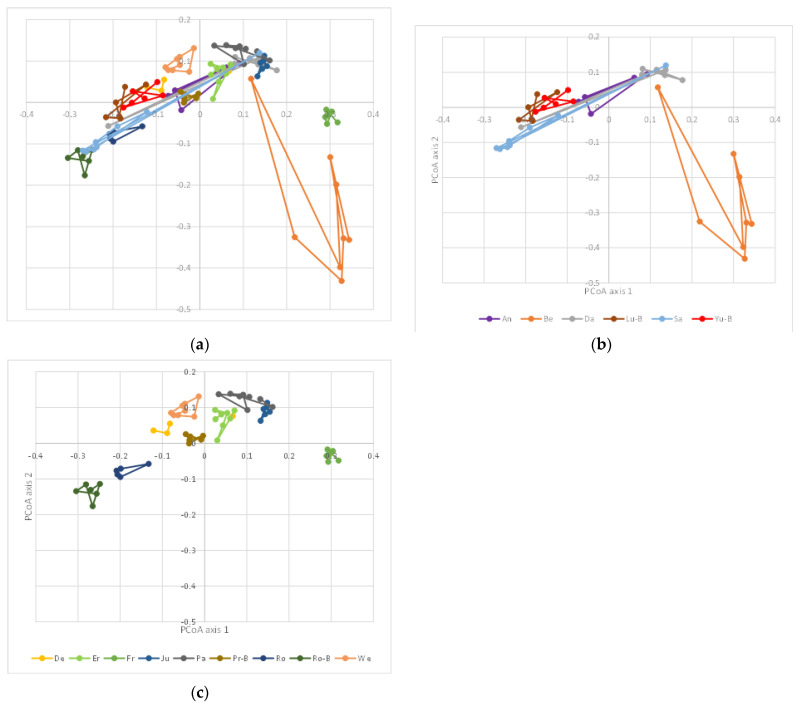
Two first axes of the unweighted PCoA (similar to Figure 1a) for all individuals (**a**); two first axes of the unweighted PCoA for those individuals with relatively large changes (**b**); two first axes of the unweighted PCoA for those individuals with more resilient microbiota (**c**). Samples are color-coded according to the different individuals (see legends of (**b**,**c**)). Axis 1 explains 18.9% and axis 2 8.5% of the variability between samples, respectively.

It is well-known and has recently been confirmed that the gut microbiota is highly individual-specific [32]. This interindividual difference may also be the reason why a dietary intervention may show an effect or not [33]. We studied the differences in β-diversity between individuals (Figure 4a) in more detail and, when studying the first two principal coordinate axes, we observed that the differences in β-diversity between the samples were either large (usually the consequence of a single intervention; Figure 4b) or very small (Figure 4c). The latter indicated a resilient gut microbiota that was not easily prone to changes [34].

**Figure 5:**
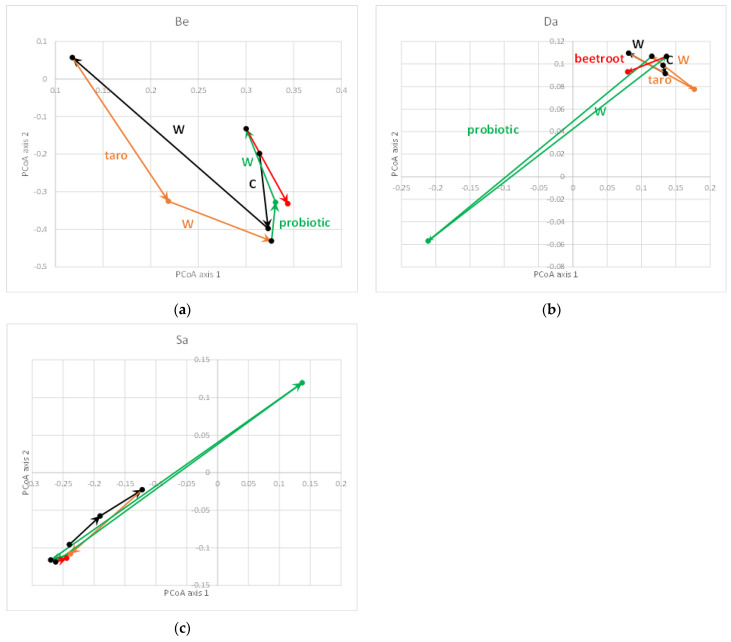
Trajectories of three individuals from Figure 4b with the largest changes. Individual Be (**a**) with large changes during the wash-out (W) period after the run-in period (C); (**b**,**c**) individuals Da and Sa, respectively, with large changes during the probiotic treatment. Samples are color-coded as follows: black: run-in period and corresponding wash-out (all baseline datapoints are colored black as well assuming that the microbiota went back to baseline after the wash-out); orange: taro treatment and corresponding wash-out; green: taro + probiotic treatment and corresponding wash-out; red: taro + beetroot treatment. Arrows indicate the sequence of treatments and the trajectory of the β-diversity changes. Axis 1 explains 18.9% and axis 2 8.5% of the variability between samples, respectively.

For the three individuals with the largest changes, the trajectory of the samples along the first two axes of the unweighted UniFrac PCoA are provided in Figure 5. In one individual (Be), the largest change was observed during the wash-out (W) period after the run-in period (C). In this individual, every treatment led to relatively large changes (in comparison with the other individuals). For the other two individuals (Da and Sa), the large change in β-diversity was caused by the addition of the probiotic to the diet whereas after the wash-out period, the microbiota composition was again similar to the other samples. The other samples of these individuals clustered more closely together, as seen for all samples for all individuals in Figure 4c. However, as can be observed in Figure 5b,c, the direction of the change in these two individuals was the complete opposite. No consistent changes between the two individuals were observed (not shown). In both individuals, *Lactobacillus* (to which *Lactiplantibacillus* in the Greengenes database belongs) increased by a factor of 1.5 (from 1.6% to 2.4% relative abundance (RA)) in individual Da and a factor of ~10 (from 1% to 9.6% RA) in individual Sa; this clearly was not reflected in the change in β-diversity as these were in an opposite direction. Moreover, the increase in *Lactobacillus* upon probiotic feeding was not consistent for other individuals, e.g., individual An showed a large decrease from 12.1% to 0.3% after the taro + probiotic treatment. In other individuals, there was no change in this taxa (e.g., 0% and 0% for individual Be (with another trajectory during the probiotic treatment compared with Da and Sa; Figure 5a), 1% and 1% for individual Er, and 22.1% and 20.7% for individual Ju, respectively).

## 4. Discussion

As it has been shown that the microbiota is involved in health and disease, including T2D [3,4,5,6], the modulation of the gut microbiota has gained enormous interest. The human gut is believed to be colonized by 250 to more than 1000 bacterial species [35,36,37] but the exact number of species in the digestive system or shared among individuals has not been determined. Several studies have shown that the microbiota is individual-specific [36,38], which may be a confounding factor in discovering the effects of (functional) food (ingredients) on the gut microbiota modulation. Researchers have been trying to define a core microbiota [36,39,40], defined as a species present in most people; however, upon increasing the population, the number of species carried by everybody reaches zero: in one study, it was observed that 57 species were present in 90% of 124 individuals but only 18 species were present in all 124 participants [36]. However, no species are shared when several cohorts are combined (our own unpublished observations). Changes in the gut microbiota occur quickly upon large changes in dietary intake such as changing from a primarily animal-based diet to a primarily plant-based diet; *Prevotella* has been stimulated with plant-based and *Bacteroides* with animal-based diets [41,42]. In a validated in vitro model of the colon, we have previously shown that changes can be observed within a period of 3 days [43] and even within 24 h [44].

The changes observed in the studies referred to above were induced by major changes in the diet or by adding a non-digestible dietary component at a relatively high dose. In the current study, 50% of wheat starch was replaced by taro starch and a probiotic or beet juice were added. Although in rodent trials we have shown the effects of taro starch on the rat microbiota [13,14,45], replacing 50% of the wheat starch with 50% of taro starch in our human volunteers did not lead to large changes in the gut microbiota with only *Butyricimonas* being affected. This was largely due to the high interindividual variation of the microbiota in the current study whereas in our rat studies, all animals had a very similar microbiota composition at the start of the trial. Several species of *Butyricimonas* have been isolated from the human gut [46,47] and they have been shown to produce butyrate. It has been frequently observed that butyrate is produced when starch is fed to the gut microbiota [48,49]. Whether or not *Butyricimonas* is capable of fermenting taro starch remains to be determined.

The effects of probiotics on the microbiota composition are also usually limited, as reviewed by Sanders [50]. Nevertheless, despite this, probiotics may affect the gut microbiota activity [51,52]. Recently, a study feeding a *Bacillus* probiotic with a dipeptide showed very few changes in the microbiota composition but increased the production of butyrate [53]. It would be interesting to study the latter using a metabolite analysis and meta-transcriptomics in future experiments. 

Although the microbiota is considered to be generally stable during adulthood [54,55], here we showed that for a few individuals the microbiota was more resilient to the imposed interventions than for other individuals. Therefore, to study the usually small effects of dietary components, it may be wise to stratify the volunteers with respect to the microbiota composition. Alternatively, the composition of the microbiota at the baseline could lead to a more personalized intervention strategy rather than attempting a compound that would be ‘fit-for-all’.

A strong point of this study is that it was a cross-over study, which allowed for a comparison of the microbiota within individuals. Despite this, the study has several limitations. Apart from the functional food interventions, the volunteers were part of a weight loss program and received advice from registered dieticians. However, because the study was cross-over, any effects that may have been induced by the weight loss program would be present in all the research arms. Given that we saw very few changes, it seems logical to conclude that the weight loss program did not lead to major changes in the composition of the gut microbiota. Apart from fasting blood glucose, the participants were characterized on a number of other metabolic syndrome-related parameters. These will be reported elsewhere. We have included IL-6 and HOMA-IR here to indicate that the metabolism of the participants was indeed disturbed. Our study population contained a greater number of female (*n* = 9) than male (*n* = 6) participants; it is believed that insulin-resistant-related cardiometabolic disorders tend to be more common in males than females, but in Indonesia, obesity and metabolic syndrome is more frequent in females [56,57]. Several studies have found a different microbiota based on sex [58] or age [59]. In our study population, the sample size may have been too small to observe these differences.

## 5. Conclusions

The highly interindividual variability in the response of the gut microbiota of the participating individuals in the study towards the effect of functional foods clouded the gut microbiota modulation effects of these functional foods. Only *Butyricimonas* was shown to be different between the control and taro ingestion (alone, or with the probiotic, or beetroot). The individuals could be divided into those with a more resilient microbiota and those with a microbiota that was more prone to changes. This interindividual difference in the microbiota compositional changes is a confounding factor in nutritional research and perhaps we should endeavor to stratify individuals based on their baseline microbiota composition in future research.

## Figures and Tables

**Table 1:** Subject characteristics and weight loss in each individual period as well as the complete intervention study.

Code	Sex	Age	Weight Control Period		Weight Difference Control	Taro Period		Weight Difference Taro	Taro + Probiotic Period		Weight Difference Taro + Probiotic	Taro + Beetroot Period		Weight Difference Taro + Beetroot	Total Weight Loss
Da	F	60	66	63.5	−2.5	65	63	−2	64	61	−3	64	62	−2	−4
Be	F	47	80	75	−5	76	72	−4	72.5	69	−3.5	71	69	−2	−11
Er	F	53	77	72	−5	73	67	−6	69	68	−1	69.5	66	−3.5	−11
Pa	M	41	86	82	−4	85	83	−2	82.5	82	−0.5	83	82	−1	−4
We	F	48	61	57	−4	59	57	−2	58	57	−1	59	57.5	−1.5	−3.5
Sa	F	45	80	76	−4	76	73	−3	76	72	−4	76	75	−1	−5
Ju	M	44	101	94	−7	101	100	−1	103.5	97.5	−6	101	98	−3	−3
Ro	F	43	60	55	−5	56.5	55.5	−1	55.5	55	−0.5	55	55.5	0.5	−4.5
An	F	44	65	63	−2	65	62	−3	63.5	63	−0.5	63	60	−3	−5
De	F	48	58	53	−5	55	53	−2	54	52	−2	54	51	−3	−7
Fr	F	36	78	76	−2	78	76	−2	79	77	−2	79	74	−5	−4
Pr	M	62	67	67	0	67	66.5	−0.5	65	64	−1	65	64	−1	−3
Yu	M	41	72.5	72.5	0	72.5	71.5	−1	73	70.5	−2.5	72	72	0	−0.5
Ro	M	33	68	66	−2	67	65.5	−1.5	66	65	−1	66	64	−2	−4
Lu	M	61	76.5	76.5	0	76.5	75.5	−1	76.5	76	−0.5	76	75.5	−0.5	−1

## Data Availability

The data are available upon reasonable request from the corresponding author. The raw sequences and corresponding meta-data will be archived in the Sequence Read Archive (SRA) repository at the NCBI upon acceptance of the manuscript.

## Supplementary Materials

The following supporting information can be downloaded at: https://www.mdpi.com/article/10.3390/nu14040781/s1, Figure S1: Difference in Butyricimonas between 4 baseline samples and the different intervention samples; Figure S2: Relative abundance of the top 25 taxa in the population. The remaining taxa are clustered under ‘Other’. A. Individual samples. B. Average of all samples of each individual.
