# Supplementary material for: Effect of Different Functional Food Supplements on the Gut Microbiota of Prediabetic Indonesian Individuals during Weight Loss
Source: Nutrients. 2022 Feb 13;14(4):781. doi: 10.3390/nu14040781 (PMC8875853; doi:10.3390/nu14040781)
Supplement: Supplementary file 1 [file nutrients-14-00781-s001.zip › nutrients-1501712-supplementary.pdf]

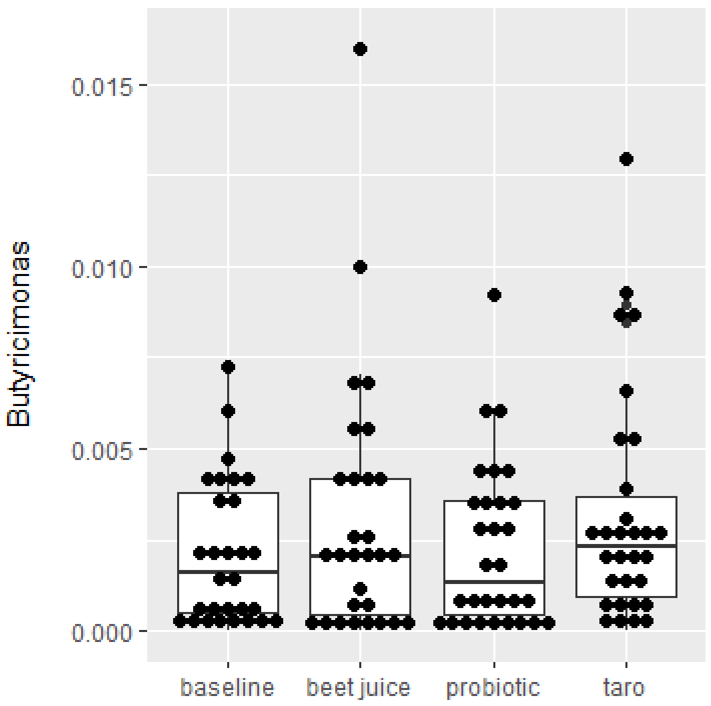

Supplementary Figure S1. Difference in *Butyricimonas* between 4 baseline samples and the different intervention samples.

A

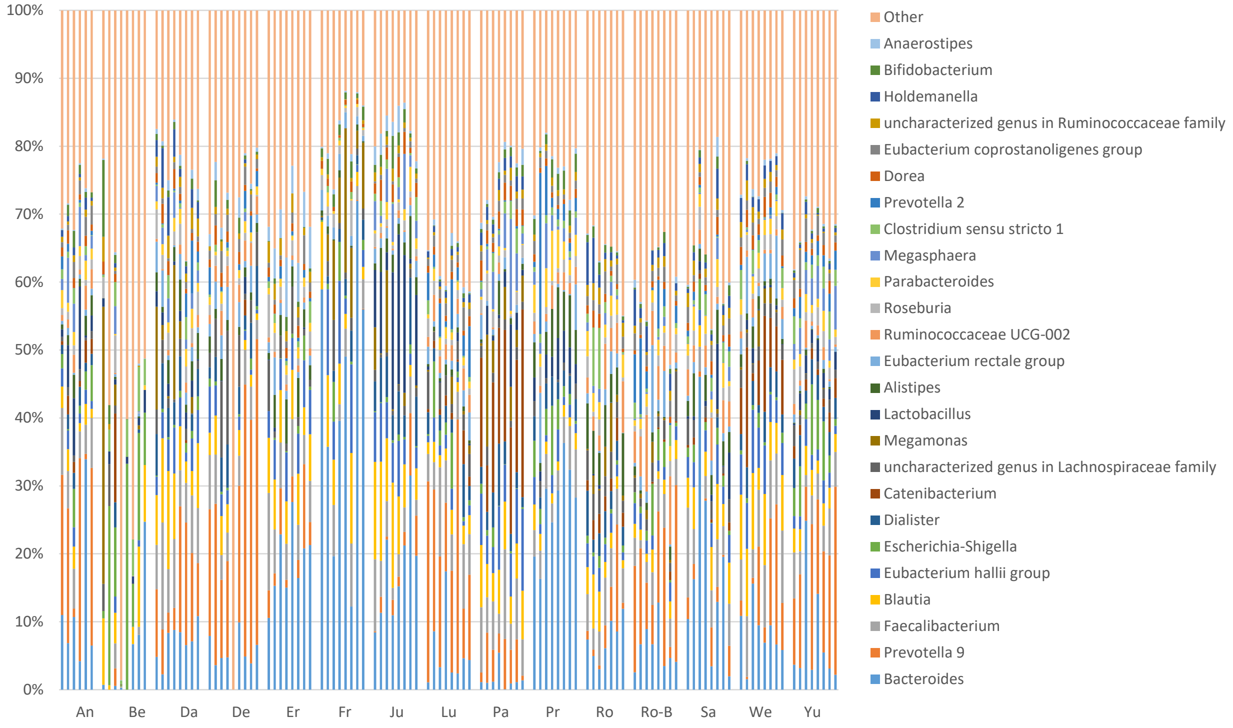

B

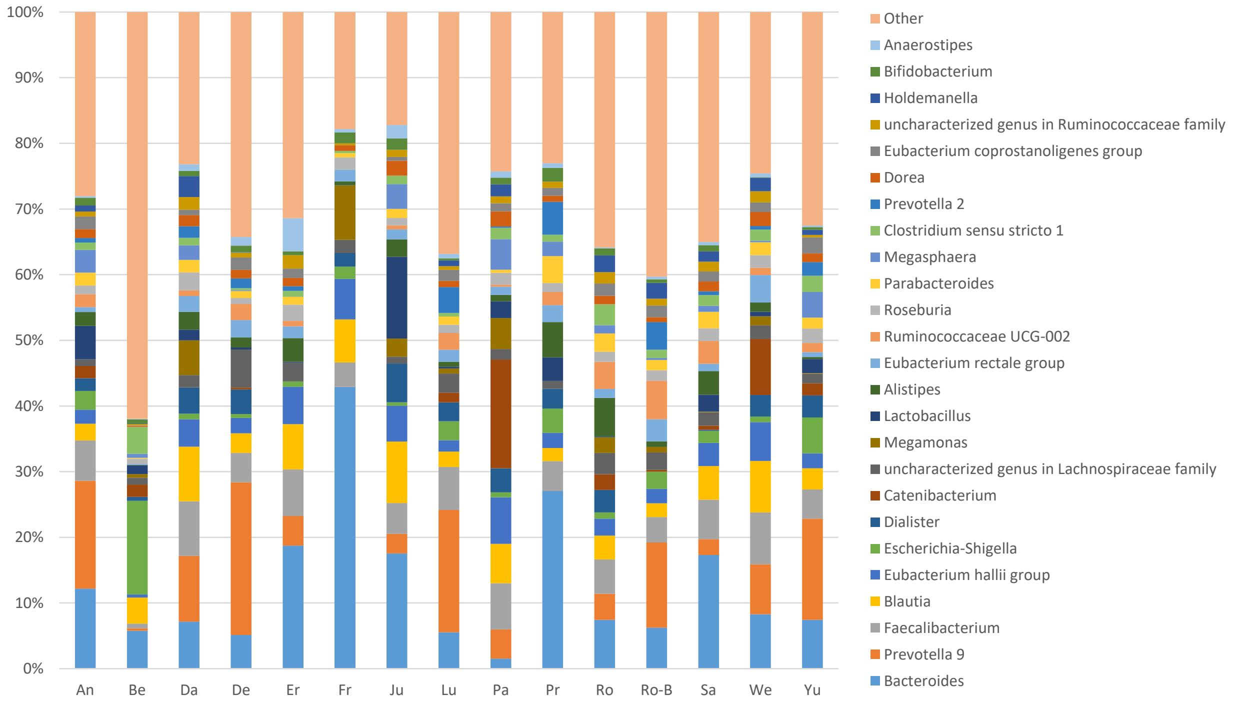

Supplementary Figure S2. Relative abundance of the top 25 taxa in the population. The remaining taxa are clustered under 'Other'. A. Individual samples. B. Average of all samples of each individual.
